# Association of lipid rafts cholesterol with clinical profile in fragile X syndrome

**DOI:** 10.1038/s41598-022-07064-z

**Published:** 2022-02-21

**Authors:** Amanda Toupin, Sérine Benachenhou, Armita Abolghasemi, Asma Laroui, Luc Galarneau, Thamàs Fülöp, François Corbin, Artuela Çaku

**Affiliations:** 1 Department of Biochemistry and Functional Genomics, Université de Sherbrooke, Sherbrooke, QC Canada; 2 Research Center on Aging, CIUSSS de l’Estrie-CHUS, Sherbrooke, QC Canada; 3 Department of Medicine, Université de Sherbrooke, Sherbrooke, QC Canada

**Keywords:** Metabolomics, Lipoproteins, Membrane lipids, Cognitive neuroscience, Development of the nervous system, Social behaviour, Synaptic plasticity, Synaptic transmission, Neurodevelopmental disorders, Predictive markers

## Abstract

Fragile X syndrome (FXS) is the most prevalent monogenic cause of intellectual disability and autism spectrum disorder (ASD). Affected individuals have a high prevalence of hypocholesterolemia, however, the underlying mechanisms and the clinical significance remains unknown. We hypothesized that decrease in the plasma cholesterol levels is associated with an alteration of cholesterol content within the lipid rafts (LRs) which ultimately affects the clinical profile of FXS individuals. The platelets LRs were isolated by ultracentrifugation on sucrose gradient from 27 FXS and 25 healthy controls, followed by measurements of proteins, cholesterol, and gangliosides content. Autistic and adaptive behaviour of affected individuals were respectively assessed by the Social Communication Questionnaire and Adaptive Behavior Assessment System. Our results suggest a decrease in the cholesterol content of LRs in FXS individuals as compared to controls. As opposed to controls, LR cholesterol was significantly associated with plasma total cholesterol (r = 0.47; *p* = 0.042) in the FXS group. Furthermore, the correlation between LRs cholesterol and the clinical profile showed a significant association with autistic traits (r = − 0.67; *p* < 0.001) and adaptative behavior (r = 0.70; *p* < 0.001). These results support the clinical significance of LR cholesterol alterations in FXS. Further studies are warranted to investigate the implication of LRs in FXS pathophysiology and ASD.

## Introduction

Fragile X Syndrome (FXS), an X-linked neurodevelopmental disorder, is the most prevalent monogenic cause of inherited intellectual disability (ID) and autism spectrum disorder (ASD)^1^. In most cases, the mutation results from a large expansion of CGG repetitions in the 5’ untranslated region of the fragile X mental retardation 1 (*FMR1*) gene leading to a deficit of the Fragile X Mental Retardation Protein (FMRP), an ubiquitary RNA-binding protein highly expressed in the brain^2,3^. FMRP controls the translation of many mRNAs encoding the proteins critical for synaptic structure and functions^4^. Studies in *fmr1* knockout (KO) mice have shown that FMRP deficiency triggers: an upregulation of signaling pathways related to the group 1 metabotropic glutamate receptor (mGluR1 and mGluR5) activation^5,6^; an excessive internalization of α-amino-3-hydroxy-5-methyl-4-isoxazolepropionic acid receptor (AMPAR); and a reduction of N-methyl-D-aspartate receptors (NMDAR)^7,8^. All the mentioned factors contributes to exaggerated long-term depression (LTD), a hallmark of FXS^9,10^.

Lipid rafts (LRs) are dynamic structures enriched in cholesterol, proteins, and gangliosides that transiently move within the plasma membrane in order to facilitate a cellular response^11^. These platforms recruit important synaptic receptors including mGluRs, NMDAR, and AMPAR^12^. Indeed, LRs facilitate protein–protein and protein-lipid interactions^13^. Moreover, LRs play crucial roles in the compartmentalization of signaling molecules on the cell membrane and intracellular trafficking^14–17^. Studies in rat brain tissues have shown that cholesterol depletion alters the morphology of dendrites (reduce the number and increase the size of dendritic spines) and modulates the synaptic activity mediated by AMPAR and NMDAR^18,19^. To the best of our knowledge, no previous study has investigated the implication and correlation of the LRs cholesterol content with the cognitive dysfunction and autistic traits of the FXS affected humans.

Each individual with FXS is unique and displays variable degrees of ID, adaptive and aberrant behaviors^20^. The clinical profile of the FXS affected individuals is frequently associated with hypocholesterolemia, a condition characterized by plasma cholesterol lower than the 5th centile of a normalized population. Specifically, 90% of FXS individuals have plasma cholesterol below the 50th centile of the normalized population and up to 30% fulfill the criteria of hypocholesterolemia^21^. Our recent report suggested a decrease of proprotein convertase subtilisin/kexin type 9 (PCSK9) activity linked to an alteration of serine-phosphorylation as an underlying mechanism of FXS associated hypocholesterolemia^22^. Indeed, PCSK9 is implicated in the degradation of low-density lipoprotein (LDL) receptors, and therefore a decrease in PCSK9 activity is associated with low plasma cholesterol. Moreover, our results suggest a negative correlation between plasma cholesterol and aberrant behavior as evaluated by the Aberrant Behavior Checklist-Community (ABC-C) total score^21^. Since cholesterol is essential for cell membrane integrity, synaptic development and neurotransmitter release, a decrease of plasma cholesterol could alter the content in cholesterol of LRs and potentially contribute to the FXS physiopathology^23,24^.

In humans, brain tissue is barely accessible. Platelets have been described as a plausible surrogate to study neurons integrity in living animals and humans^25^. Platelets express many receptors and markers that are also found in neurons and thus may recapitulate the defects observed in FXS neurons, such as glutamate signaling pathways^25–28^. Moreover, FMRP levels quantified in the platelets of the FXS affected individuals are lower than those observed in the healthy controls and significantly correlate with cognitive functions^29^.

Perturbations of plasma cholesterol have been reported in several cognitive disorders such as Alzheimer^30,31^, Smith-Lemli-Opitz-Syndrome^32^, Parkinson^33^, and Huntington^31,34^. Moreover, perturbation of the cholesterol content of LRs in platelets has been revealed in neurodevelopmental and neurocognitive diseases^35–37^. However, the implication of LRs in the FXS human pathophysiology remains unclear. This study thus aims to explore the lipid content of LRs in platelets of FXS individuals as compared to healthy individuals. We also investigated the relation of LRs cholesterol with lipid profile and cognitive functions in FXS.

## Materials and methods

### Study design and population

The design and population of the LipidX study have been described previously^21^. Characteristics of FXS and control participants are shown in Supplementary Table 1. Briefly, 27 FXS individuals and 25 healthy individuals were recruited *at Centre de Recherche du Centre Hospitalier Universitaire de Sherbrooke* (CHUS), between January and June 2015. Individuals with acute conditions, disorders affecting lipid metabolism, or treated with lipid-lowering drugs were excluded. The LipidX study was approved by the Scientific and Ethics Board of the local research center. All participants provided informed consent prior to participation in the study or by the tutor/caregiver on behalf of the mentally disabled minors and adults with FXS. The study was conducted according to the guidelines of the Declaration of Helsinki, and approved by the Institutional Review Board of *Centre de Recherche du Centre Hospitalier de l’Université de Sherbrooke* (protocol code 2015-946, 14-220 and date of approval January 28th 2015).

### Clinical and metabolic evaluations

Medical history and anthropometric measures including weight, height, body mass index (BMI) were obtained for each participant. The clinical profile of each FXS participant was assessed by the following questionnaires and completed by their caregiver: (1) age-adjusted Adaptive Behavior Assessment System – Second Edition (ABAS-II); (2) Aberrant Behavior Checklist-Community (ABC-C); (3) Social Communication Questionnaire (SCQ) and (4) Anxiety, Depression, and Mood Scale (ADAMS). Fasting blood samples were collected in acid citrate dextrose (ACD) (BD vacutainer) from all participants to perform plasma lipid analyses and platelet isolation. Specifically, the following tests were performed at the clinical biochemistry laboratory of the *CIUSSS de l’Estrie-CHUS*: plasma total cholesterol (TC), triglycerides (TG), C-HDL were measured by enzymatic methods (Modular Roche P800); C-LDL was calculated using the Friedewald formula; apolipoprotein B (ApoB) and apolipoprotein A (ApoA) were determined by immunoturbidimetric assays (Roche Diagnostics, Cobas 501 analyser). FMRP quantitation was performed on platelets by Western Blot as previously described^29^.

### Lipid rafts preparation

#### Platelet isolation

Platelets were isolated from the whole blood as previously described^29^. Briefly, the platelet-rich plasma (PRP) was separated from whole blood after a 10 min centrifugation at 300 xg at room temperature and collected without disturbing the buffy coat. The platelet count was then performed on a flow cytometer analyzer (DXH-9000, Beckman Coulter®). After a 15 min centrifugation at 2,400 xg, the platelet pellet was isolated and then washed twice in phosphate-buffered saline (PBS) containing EDTA 5 mM (Invitrogen by Thermo Fisher Scientific).

#### Lipid rafts isolation

**Figure 1:**
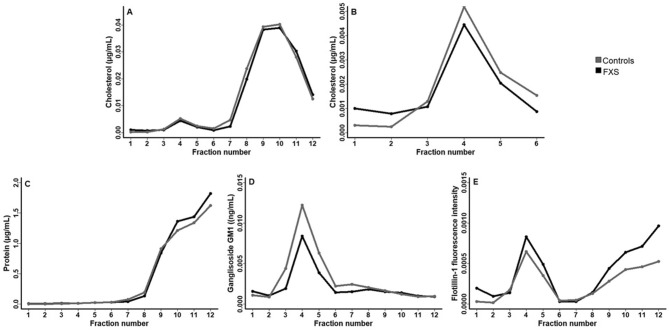
Distribution of LRs and non-LRs components in FXS and controls. Lysates of platelets extracted from controls (gray) and FXS (black) were separated on a sucrose gradient and monitored for: (**A**) cholesterol; (**B**) cholesterol from (**A**) from F1 to F6; (**C**) proteins; (**D**) gangliosides GM1; (**E**) Flotillin-1. All parameters are normalized by millions of platelets.

Lipid rafts were isolated according to previously described protocols^38,39^. Briefly, the platelet pellet was disrupted in lysis buffer (PIPES 0.05 M, EDTA 0.025 M, NaCl 0.75 M, Protease inhibitor P8340, Triton X-100 1%) for 30 min and homogenized using a syringe and needle 27G (Terumo). The platelet lysate was mixed with 2 mL of 80% sucrose followed by two 4 mL layers containing respectively 30% and 5% sucrose to allow a gradient formation during the centrifugation. The lysate was centrifuged at 37,000 RPM for 18 h at 4 °C (Beckman Optima LE-80 K, Beckman Coulter, Fullerton, CA with the rotor SW40, Beckman Coulter). After the centrifugation, 12 fractions of 1 mL were gently collected from the top (F1) through to the bottom layer (F12). The enriched LR fractions were identified by monitoring the elution of flotillin-1 by Western Blot. Briefly, 10 µL of each fraction were separated on a 4–10% gradient SDS polyacrylamide gel and proteins were transferred on nitrocellulose. Membranes were blocked in 5% non-fat dry milk, incubated with anti-flotillin-1 (1:1,000) followed by anti-rabbit IgG coupled to Alexa FluorVR 680 (1:10,000). Fluorescence was revealed using an Odyssey Infrared Imaging System (LI-COR Biosciences). Semi-quantification of flotillin-1 was performed with Image-J software (Fig. 1, Supplementary Figs. 1A and 2)^40,41^. Since LRs are enriched in flotillin-1 and ganglioside GM1, the overlap in flotillin-1 expression and ganglioside GM1 abundance was used as the criteria to identify LR fractions (Supplementary Figs. 1–3). The LR fractions were kept frozen at –80 °C before further analyses.

#### Lipid raft components measurement

Cholesterol measurement was performed using the Amplex® Red Cholesterol Assay Kit (Life Technologies). LRs fractions were diluted 4 times in the reaction buffer and 50 µL were used for the assay. Fifty microliters of working solution (300 µM Amplex Red reagent, 2 U/mL HRP, 2 U/mL cholesterol oxidase, 0.2 U/mL cholesterol esterase) was added to the samples. After an incubation of 30 min, at 37 °C in the dark, the fluorescence was measured at 560 nm in a microplate reader (PR 3100 TSC, Bio-Rad). Dot-blot was used to measure gangliosides: 200 µL of the standards Ganglioside GM1, Asialo (Santa Cruz) (0.000; 0.313; 0.625; 1.250; 2.500; 5.000 ng/mL) and samples (diluted 5 times) were loaded on nitrocellulose. Each dot was washed three times with PBS, blocked with 5% non-fat dry milk in PBS for 1 h at room temperature, washed twice with PBST (PBS with 0.05% Tween 20) and incubated overnight at 4 °C on a shaker with 1 µg/mL cholera toxin B/horseradish peroxidase conjugate (HRP-CTB) (Molecular Probes, Thermo Fisher) in 5% non-fat milk. The following day, membranes were washed twice with PBST and once with PBS then revealed using an enhanced chemiluminescence (ECL) detection kit on a ChemiDoc system (Bio-Rad) (Supplementary Fig. 1B and 3)^38^. Protein concentration was determined using the Micro BCA assay kit as described by the manufacturer (Pierce 2020).

### Statistical analyses

Descriptive analyses were performed on demographic variables. Depending on the normality distribution assessed by the Shapiro–Wilk test, the Mann–Whitney U test was used for the comparison between the two groups. The Pearson correlation coefficient (r), univariate linear, and multiple regressions were respectively performed to investigate the association and to predict clinical profile. Statistical significance was established at an alpha level of rejection of 0.05. Multiple testing correction was performed using FDR (Benjamini and Hochberg). Outliers were removed from the analyses according to the ROUT method (Q = 1%) in Graphpad Prism software version 9 (La Jolla, CA, USA)^42^. Other statistical analyses and graphics were performed in R (version 4.0.5, CRAN).

## Results

### Distribution of the lipid rafts components

The average distribution of LRs components including cholesterol, proteins, and gangliosides GM1, all adjusted to obtain concentrations expressed in one million platelets are shown in Fig. 1. The same pattern of distribution is obtained for both controls and FXS supporting technical consistency in the preparation. Specifically, cholesterol concentration has a bimodal distribution with the first peak in fractions F3 to F5 (Fig. 1B) and the second peak in bottom fractions, F8 to F12 (Fig. 1A). The proteins are more concentrated in bottom fractions F8 to F12. (Fig. 1C), while gangliosides GM1 showing a peak in top fractions F3 to F6 (Fig. 1D). The combined fractions 2 to 6 were referred as LRs according to the overlap of flotillin-1 enrichment and high gangliosides GM1 content (Fig. 1E). LRs fractions were associated with the first peak of cholesterol distribution (Supplementary Figs. 1–3).

Overall, FXS individuals have lower LRs cholesterol and ganglioside GM1 as compared to controls: 0.005 *vs* 0.007 (µg/mL)/million platelets, and 0.013 *vs* 0.019 (ng/mL)/million platelets, respectively, although the differences are not statistically significant (Fig. 1). Total cellular protein, cholesterol and ganglioside GM1 content obtained from all fractions summed together do not show a significant difference between FXS and controls.

### Lipid raft cholesterol and clinical profile

**Figure 2:**
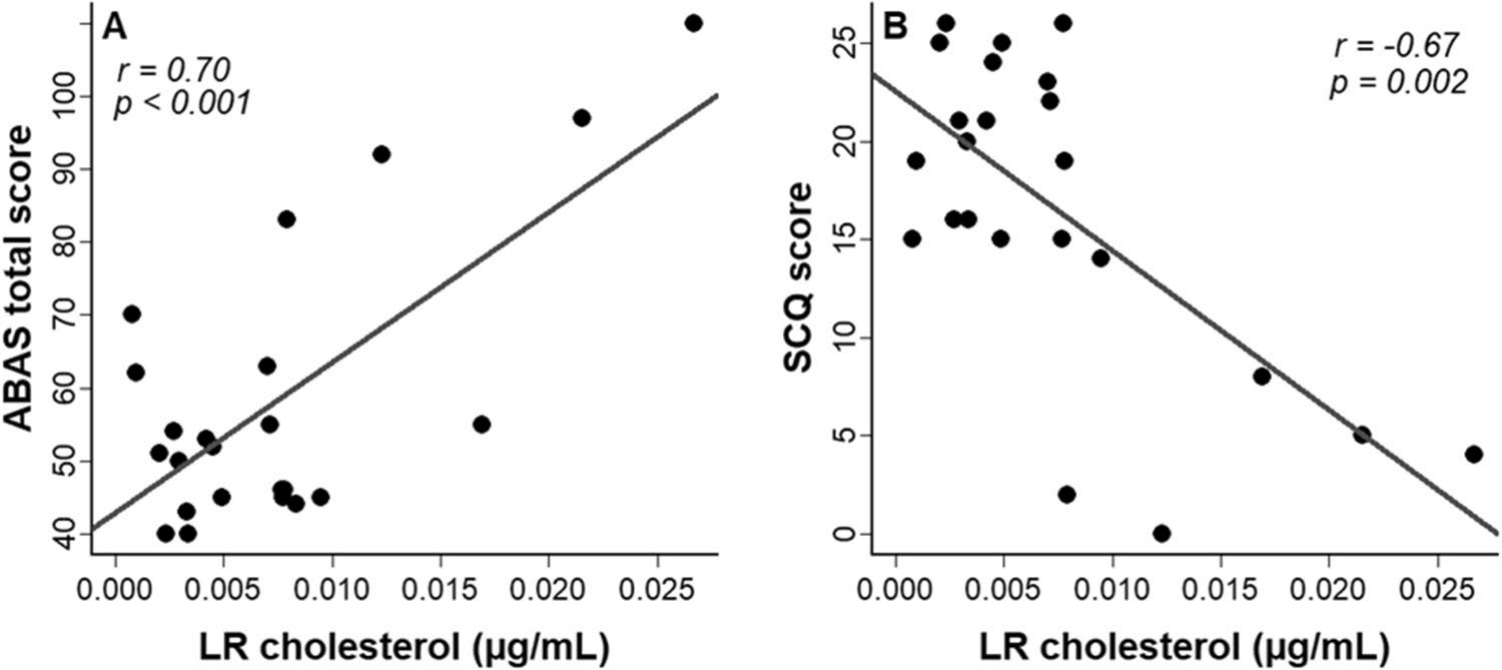
Association of LRs cholesterol with (**A**) ABAS total score and (**B**) SCQ score. r: Pearson correlation coefficient.

LRs cholesterol levels of FXS affected individuals positively correlate with ABAS total score (r = 0.70; *p* < 0.001) (Fig. 2A) as well as with three ABAS subdomains: conceptual (r = 0.67; *p* < 0.001), social (r = 0.66; *p* = 0.001) and practical (r = 0.62; *p* = 0.004). Furthermore, LRs cholesterol significantly correlates with SCQ score (r = − 0.67; *p* = 0.002) (Fig. 2B). Specifically, individuals with lower cholesterol have lower adaptative functions as shown by ABAS scores and more ASD symptoms as shown by SCQ score. We also performed multiple linear regressions adjusting for age, body mass index, and FMRP (absence or presence) to explore the effect of LRs cholesterol on ABAS and SCQ scores. The results are still significant for ABAS total score, ABAS conceptual and social subscales as well as SCQ score (Supplementary Table 2). However, LRs cholesterol is not associated with ADAMS and ABC-C scores.

### Associations between lipid rafts cholesterol and lipid profile

**Figure 3:**
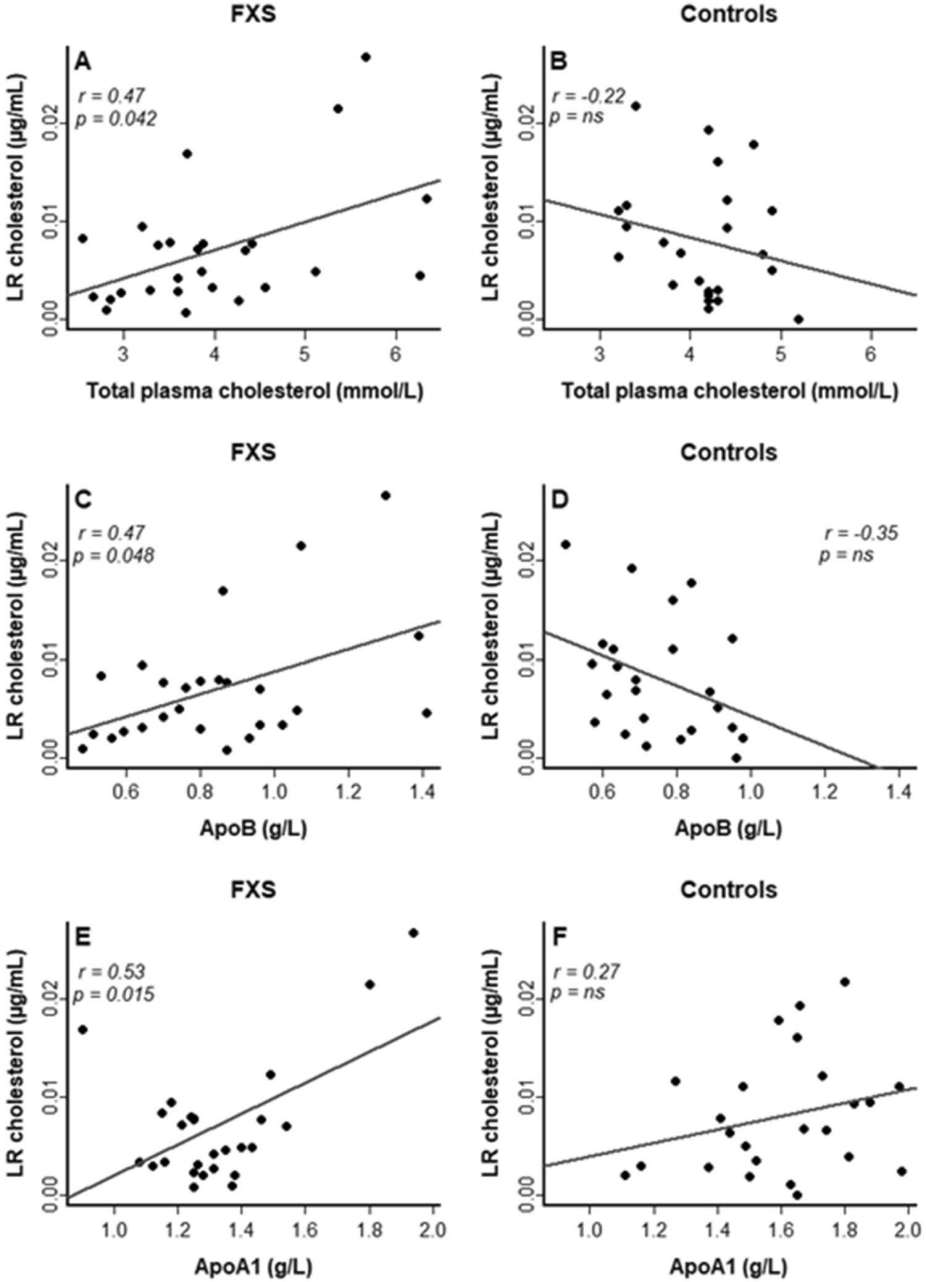
Association of LRs cholesterol with (**A**) plasma total cholesterol in FXS; (**B**) plasma total cholesterol in controls; (**C**) ApoB in FXS; (**D**) ApoB in controls; (**E**) ApoA1 in FXS and (**F**) ApoA1 in controls. r: Pearson correlation coefficient.

Associations between LRs cholesterol and lipid profile are shown in Fig. 3. In the FXS cohort, LRs cholesterol positively correlates with plasma total cholesterol (r = 0.47, *p* = 0.042), LDL-C (r = 0.49, *p* = 0.039), ApoB (r = 0.47, *p* = 0.048), and ApoA1 (r = 0.53, *p* = 0.015). Meanwhile, no significant correlations between these parameters are observed in the control cohort. In addition, no significant correlation is obtained between total cell cholesterol (sum of all fractions) and plasma total cholesterol, in neither FXS affected individuals, nor in control healthy individuals.

## Discussion

Lipid rafts are plasma membrane microdomains rich in cholesterol that are important for signal transduction. In neurons, LRs serve as ordered platforms that coordinate signaling molecules through membrane receptors and are involved in synaptic transmission. LRs from human platelets have been investigated in cognitive disorders such Alzheimer disease^43^. Indeed, platelets have been used as a surrogate to study neuron function of human brain in several neurocognitive and neurodevelopmental disorders including Alzheimer disease or ASD^43,44^. The present study reports the first investigation of LRs from platelets of FXS participants as compared to healthy controls.

Our data show a slight reduction of LRs cholesterol in FXS as compared to healthy controls. Although the difference was not statistically significant, the direct correlation of LRs cholesterol with adaptative functions suggests clinical importance. Specifically, LRs cholesterol correlated positively with adaptative behavior as evaluated by ABAS: FXS participants with lower LRs cholesterol displayed a lower degree of three adaptive domains including practical (r = 0.62), social (r = 0.66), and conceptual skills (r = 0.67). In a previous study, we reported a positive link between plasma cholesterol and ABAS practical domain (r_s_ = 0.49)^21^, while the present study reports a stronger association between LRs cholesterol and all adaptive domains. In addition, lower LRs cholesterol was associated with a higher score of autistic traits as evaluated by the SCQ questionnaire. These data agree with our previous report in ASD population that showed an increase of cognitive impairment risk when the plasma cholesterol is below the 10th centile of normalized population^45^. Taken together, these findings suggest a potential mechanistic implication of low cholesterol in cognitive dysfunction of FXS individuals probably through disruption of cholesterol content in LRs.

In neurons, LRs are implicated in synapse morphology and functions^18^. Particularly, LRs cholesterol is important for receptor trafficking and interaction of signaling molecules with their receptors^18^. A study in *fmr1* KO mice showed a reduction of mGluR1 internalization (by caveolin-mediated pathway) after cholesterol depletion of LRs, followed by a higher expression of mGluR1 in hippocampus neurons^46^. Another study showed that cholesterol depletion of LRs resulted in a significant reduction of NMDA receptors in cultured wild rat neurons^47^. Importantly, the excessive signaling of mGluR1 is a hallmark of FXS and a recent report suggests an association with NMDA receptor hypofunction^7^. Similarly, in vitro research suggests an alteration of platelet function following cholesterol depletion of LRs^48^. We thus hypothesize that LRs from platelets are a study model of LRs from neurons. Indeed, a reduction of cholesterol content in LRs might contribute to FXS pathophysiology and ultimately to clinical profile.

Considering the high prevalence of hypocholesterolemia in FXS and ASD as well as the reported link of plasma cholesterol with clinical profile, we explored whether plasma level of cholesterol is associated with cholesterol content in LRs^21,45^. Our results show a significant direct association between the plasma total cholesterol (including ApoB and ApoA1 containing lipoproteins) and LRs cholesterol only in the FXS group. In contrast, no significant association is obtained between plasma total cholesterol and total cell cholesterol in neither FXS nor control group. We believe thus that abnormal distribution of membrane cholesterol into lipid rafts might be the underlying cause of low cholesterol content in LR, observed in FXS. Indeed, cell cholesterol is determined not only by in situ synthesis but also by cellular influx and efflux mediated by LDL and HDL respectively^49^. ApoB represents lipoproteins (mainly LDL) delivering hepatic cholesterol to the peripheral tissues, while ApoA1 (mainly HDL) mediates the reverse transport of cholesterol from peripheral cells to the liver^49–52^. The cholesterol distribution into lipid rafts is regulated by Apolipoprotein A-I binding protein (AIBP)^53^. The association of AIBP with ApoA1 or HDL-C allows a rapid and effective efflux of cholesterol through an interaction with ATP-binding cassette transporter type A1 (ABCA1) or through cytoskeleton changes^54^. In vitro and in vivo animal studies suggest that high HDL levels are associated with low LRs cholesterol content. However, AIBP does not promote cholesterol efflux in the absence of ApoA1 or HDL^53^. The finding that FXS had significantly lower HDL-C compared to healthy controls might explain the direct link observed between ApoA1 and LR cholesterol in FXS group, whereas correlation results in our control group did not corroborate with previous in vitro findings^55^. The limited number of participants or the cellular model used in the present study (platelets) might account for this discrepancy. However, this different interaction of cholesterol pools in FXS as compared as controls highlight the possibility of an impairment of cholesterol regulation in LRs. Further studies with labeling technics are warranted in FXS to validate the hypothesis that an alteration of control mechanisms of cholesterol influx and efflux alters the distribution of cholesterol into LRs^56^.

It is noteworthy to mention brain cholesterol is synthesized in situ and is independent from peripheral cholesterol metabolism^57^. Although two pools are separated by hematoencephalic barrier, the same genes are implicated in cholesterol metabolism and thus similar lipid alterations might be present in both, brain and peripheral circulation. Several studies in neurodevelopmental and neurodegenerative conditions reported associations between plasma cholesterol and cognitive or behavioral functions^57–60^. We hypothesize that brain cholesterol levels are also decreased in FXS and might trigger to some extend the clinical profile. Since FMRP regulates the expression of many cytoplasmic proteins; it might be involved in lipid synthesis and transport and its absence might dysregulate lipid rafts properties. However, further studies are needed to fully understand the interplay between FMRP and cholesterol metabolism, particularly if it regulates sterol regulatory element binding proteins.

Few study trials of lovastatin have been performed on FXS individuals, specifically short-term treatment periods showing discrepancy results of phenotype improvement^61,62^. It has been shown that lovastatin inhibits the ERK pathway in FXS resulting in a normalization of protein synthesis^28,63^. On the other hand, lovastatin is a cholesterol-lowering agent that inhibits 3-hydroxy-3-methylglutaryl coenzyme A (HMG-CoA) reductase, the rate-controlling enzyme of cell cholesterol biosynthesis^64^. It has been shown that lovastatin decreases cholesterol content in LR of dendrites of cultured hippocampal neurons and cholesterol depletion following statin treatment leads to a gradual loss of synapses^18^. In FXS, there is an abnormally high density of dendritic spines that are usually long and immature^65,66^. In the light of our results, we can hypothesize that lovastatin might lead to a decrease in the number of these abnormal spines through depletion of cholesterol content in LR. This might justify short-term treatments with lovastatin in FXS. However, we should be careful when considering long-term treatment periods with statins in these individuals, especially those with hypocholesterolemia.

Some limitations should be considered in the present study. First, although, we used platelet as a model to investigate neurons LRs, we agree that biopsies of neuronal tissue such as motor endplates or peripheral nerves might strengthen the conclusions. However, considering the invasive procedure, it would be difficult to justify of human biopsies beyond clinical necessity. As an alternative, the implication of cholesterol content in LRs in FXS pathogenesis might be further investigated in *fmr1* KO mice neurons. Second, the small sample size of each group limited us to conclude significant differences between LRs components from FXS and controls. Moreover, the small number of FXS females, limited our conclusion about the reflection of sex effect in our results. The sample size was primarily calculated to detect a difference in plasma cholesterol of the FXS population (LipidX study)^21^. A larger study of FXS individuals is warranted to validate the correlations of LRs components with lipid and clinical profiles. Third, since lipid rafts are enriched in other lipids, such as, sphingolipids or saturated fatty acids, a deeper lipidomic analysis would have improved the interpretation of our findings^67^. Fourth, since we did not have controls affected by hypocholesterolemia, it remains unclear whether our results are caused by the pathogenesis of FXS or is a consequence of hypocholesterolemia in FXS individuals for instance, demyelination due to the lack of cholesterol and leading to clinical manifestations.

In summary, this study was a first attempt to investigate composition of LRs from platelets of FXS participants, specifically the association of LRs cholesterol with cognitive functions of FXS such as adaptative behavior and autistic profile. Our results support the use of platelets as a surrogate model for the neuron to study the implication of LRs alteration in FXS. However, larger studies including lipidomic analysis of LRs are warranted to validate our results not only on FXS, but also in other neurodevelopmental disorders such as ASD. Moreover, bioinformatic studies investigating the link between *fmr1* and genes involved in the lipid metabolism might further elucidate the implication of FMRP in hypocholesterolemia pathogenesis.

## Supplementary Information

Supplementary Information.

## Data Availability

All data generated or analyzed during this study are included in this article. If any additional information is required, it may be obtained by request from the corresponding author.
