# Supplementary material for: Association of lipid rafts cholesterol with clinical profile in fragile X syndrome
Source: Sci Rep. 2022 Feb 21;12:2936. doi: 10.1038/s41598-022-07064-z (PMC8861159; doi:10.1038/s41598-022-07064-z)
Supplement: Supplementary file 1 — Supplementary Information. [file 41598_2022_7064_MOESM1_ESM.docx]

Association of Lipid Rafts Cholesterol with Clinical Profile in Fragile X Syndrome

Amanda Toupin ^1^, Sérine Benachenhou ^1^, Armita Abolghasemi ^1^, Asma Laroui ^1^, Luc Galarneau ^1^, Thamàs Fülöp ^2,3^, François Corbin ^1^, Artuela Çaku ^1,*^

1 Department of Biochemistry and Functional Genomics, Université de Sherbrooke, Sherbrooke, Québec, Canada

2 Research Center on Aging, CIUSSS de l'Estrie-CHUS, Sherbrooke, Quebec, Canada

3 Department of Medicine, Université de Sherbrooke, Sherbrooke, Quebec, Canada

* Corresponding author: Artuela.S.Caku@USherbrooke.ca

**Supplementary Table 1.** Population characteristics, lipid profile and clinical profile. Values are presented as median with interquartile 1 (IQ1) and interquartile 3 (IQ3).

| **Characteristics** | **All FXS** | **Males FXS** | **Females FXS** | **All controls** | **Male controls** | **Female controls** |
| --- | --- | --- | --- | --- | --- | --- |
| Sex (%) | 27 | 24 (88.9%) | 3 (11.1%) | 25 | 14 (56%) | 11 (44%) |
| Age (years) | 24.0  [20.5, 27.0] | 24.0  [19.8, 25.3] | 29.0  [27.5, 35.0] | 22.5  [21.3, 25.8] | 22.0  [20.4, 25.5] | 24.0  [22.0, 38.0] |
| BMI (kg/m^2^) | 26.0  [21.9, 30.6] | 25.8  [21.4, 29.1] | 33.4  [29.6, 37.4] | 22.1  [20.0, 24.0] | 23.3  [20.4, 25.5] | 21.5  [20.0, 22.5] |
| TC (mmol/L) | 3.8  [3.3, 4.4] | 3.7  [3.3, 4.3] | 5.7  [5.5, 6.0] | 4.2  [3.8, 4.4] | 4.2  [4.0, 4.7] | 4.2  [3.4, 4.4] |
| TG (mmol/L) | 0.9  [0.6, 1.5] | 0.8  [0.6, 1.4] | 1.4  [1.0, 2.7] | 0.9  [0.7, 1.2] | 1.1  [0.7, 1.7] | 0.7  [0.7, 0.9] |
| HDL (mmol/L) | 1.0  [0.9, 1.2] | 1.0  [0.8, 1.1] | 1.2  [1.1, 1.5] | 1.4  [1.3, 1.6] | 1.3  [1.2, 1.6] | 1.6  [1.4, 1.6] |
| LDL (mmol/L) | 2.3  [1.8, 2.7] | 2.2  [1.7, 2.5] | 3.4  [3.1, 3.9] | 2.3  [1.8, 2.7] | 2.5  [2.0, 2.9] | 2.1  [1.7, 2.4] |
| ApoB (g/L) | 0.9  [0.7, 1.0] | 0.8  [0.6, 0.9] | 1.3  [1.2, 1.3] | 0.7  [0.6, 0.9] | 0.8  [0.7, 1.0] | 0.7  [0.6. 0.8] |
| ApoA1 (g/L) | 1.3  [1.2, 1.4] | 1.3  [1.2, 1.4] | 1.8  [1.6, 1.9] | 1.6  [1.4, 1.7] | 1.5  [1.4, 1.7] | 1.7  [1.7, 1.8] |
| SCQ score | 19.0  [14.5, 22.5] | 19.5  [15.0, 23.3] | 4.0  [2.0, 4.5] | - | - | - |
| ADAMS | 32.0  [11.0, 37.0] | 33.5  [19.3, 38.5] | 5.0  [3.0, 8.0] | - | - | - |
| ABAS global | 52.5  [45.0, 62.3] | 51.0  [45.0, 55.0] | 97.0  [94.5, 103.5] | - | - | - |
| ABAS conceptual | 57.0  [49.0, 65.0] | 56.0  [49.0, 62.5] | 98.0  [92.5, 102.0] | - | - | - |
| ABAS social | 62.0  [57.0, 68.0] | 62.0  [55.5, 66.0] | 87.0  [82.5, 98.0] | - | - | - |
| ABAS practical | 60.0  [46.0, 76.0] | 54.0  [45.3, 66.8] | 109.0  [105.0, 110.0] | - | - | - |
| ABCC global | 41.0  [9.0, 51.0] | 42.0 [22.3, 57.8] | 4.0  [2.5, 6.5] | - | - | - |


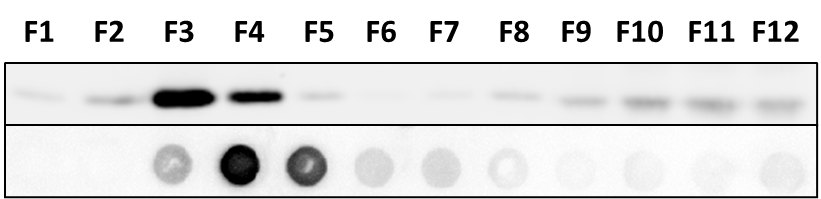


**A**

**B**

**Supplementary Figure 1.** Comparison of A) Western Blot on Flottilin-1 for each fraction separated by sucrose gradient and B) Dot Blot on ganglioside GM1 for each fraction separated by sucrose gradient. Full blots of each participant are available in Supplementary Figures 2-3.


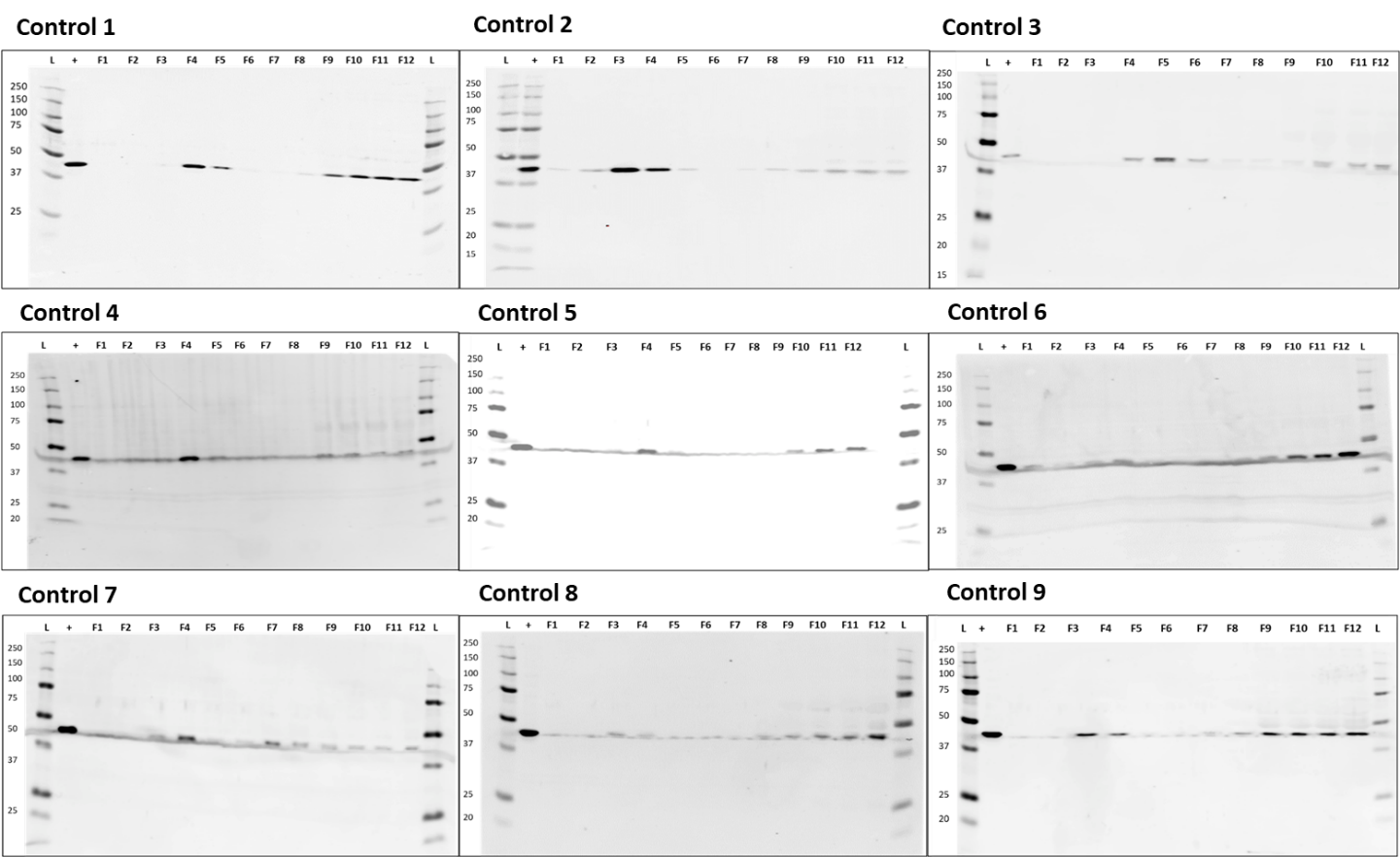


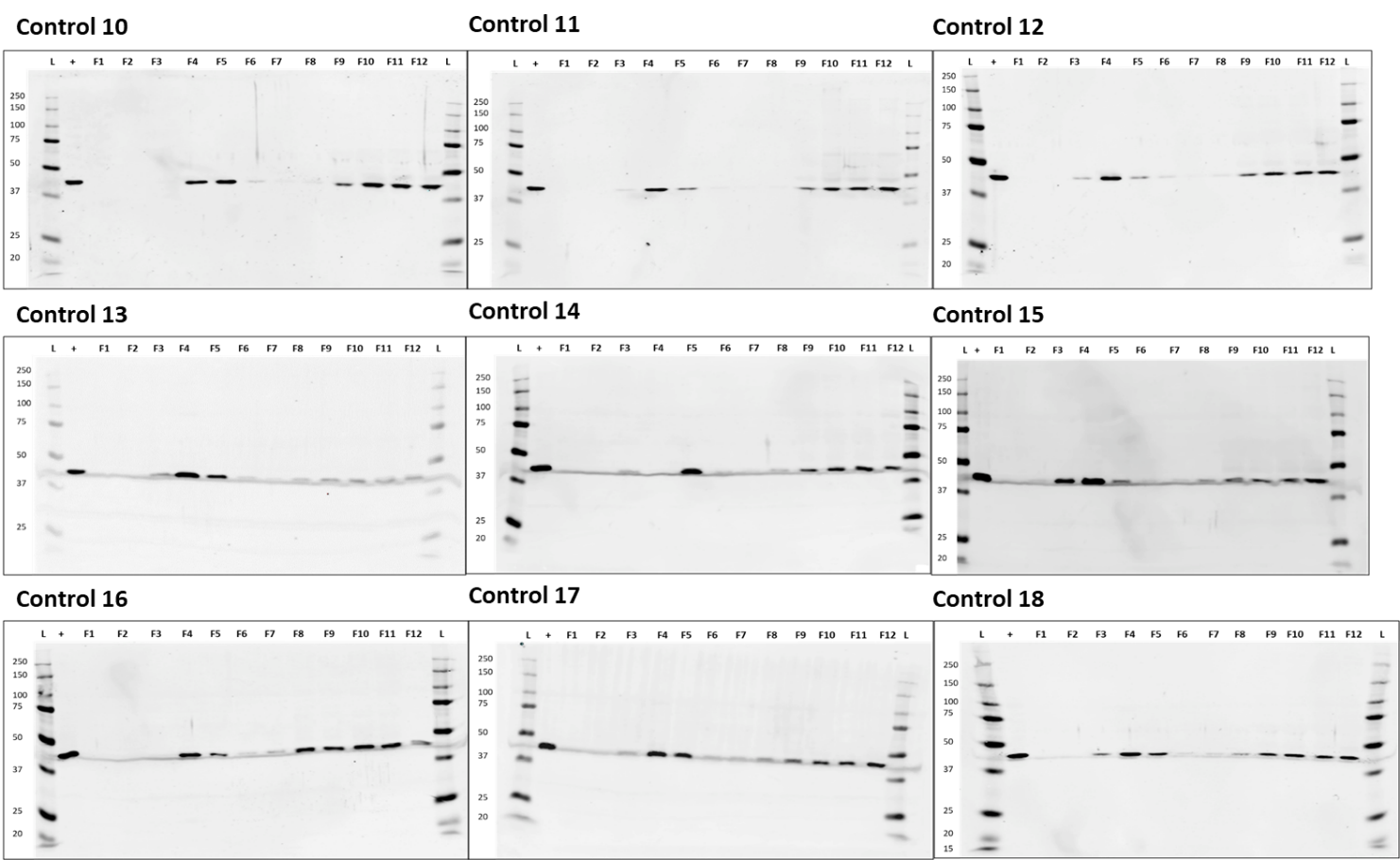


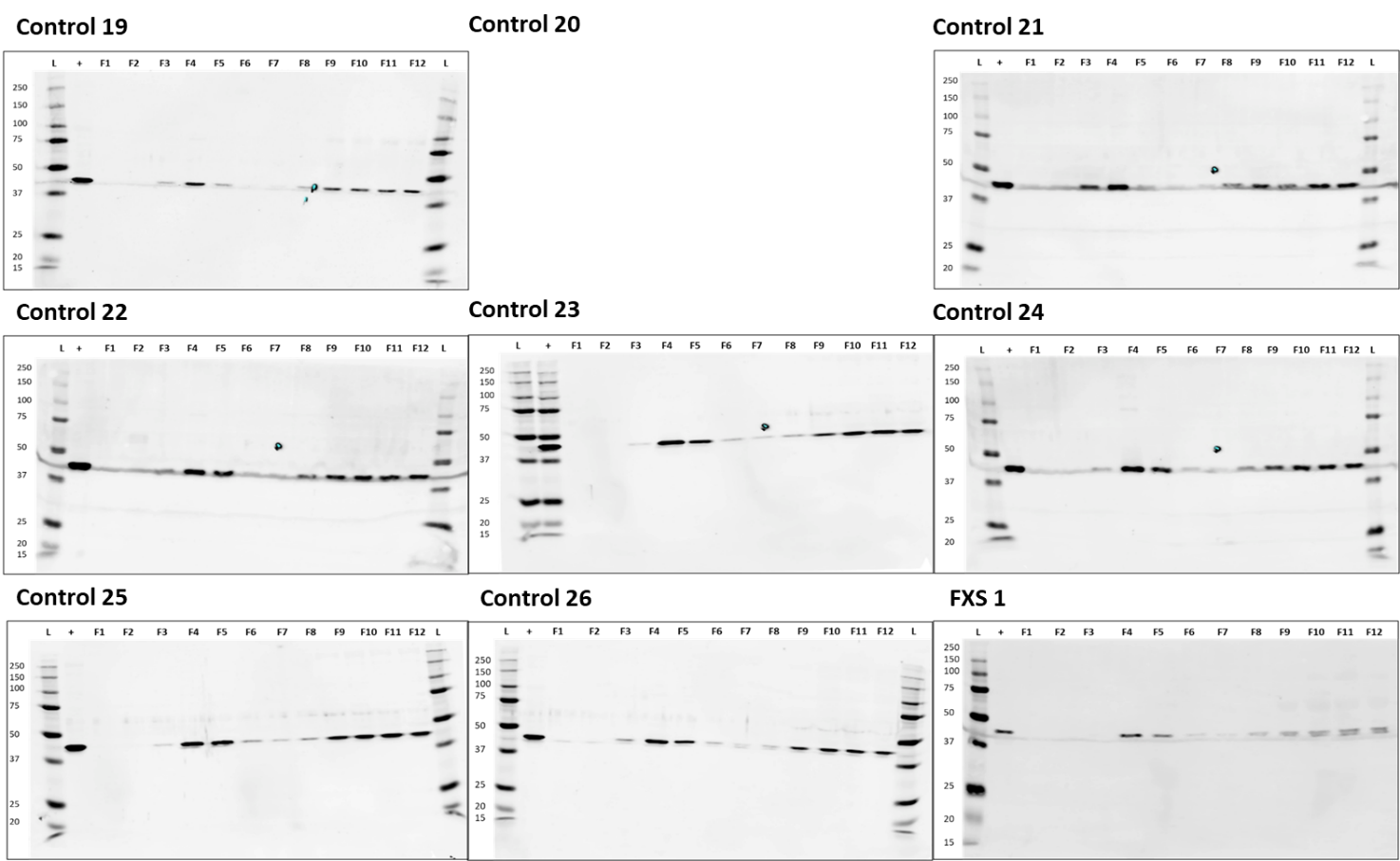


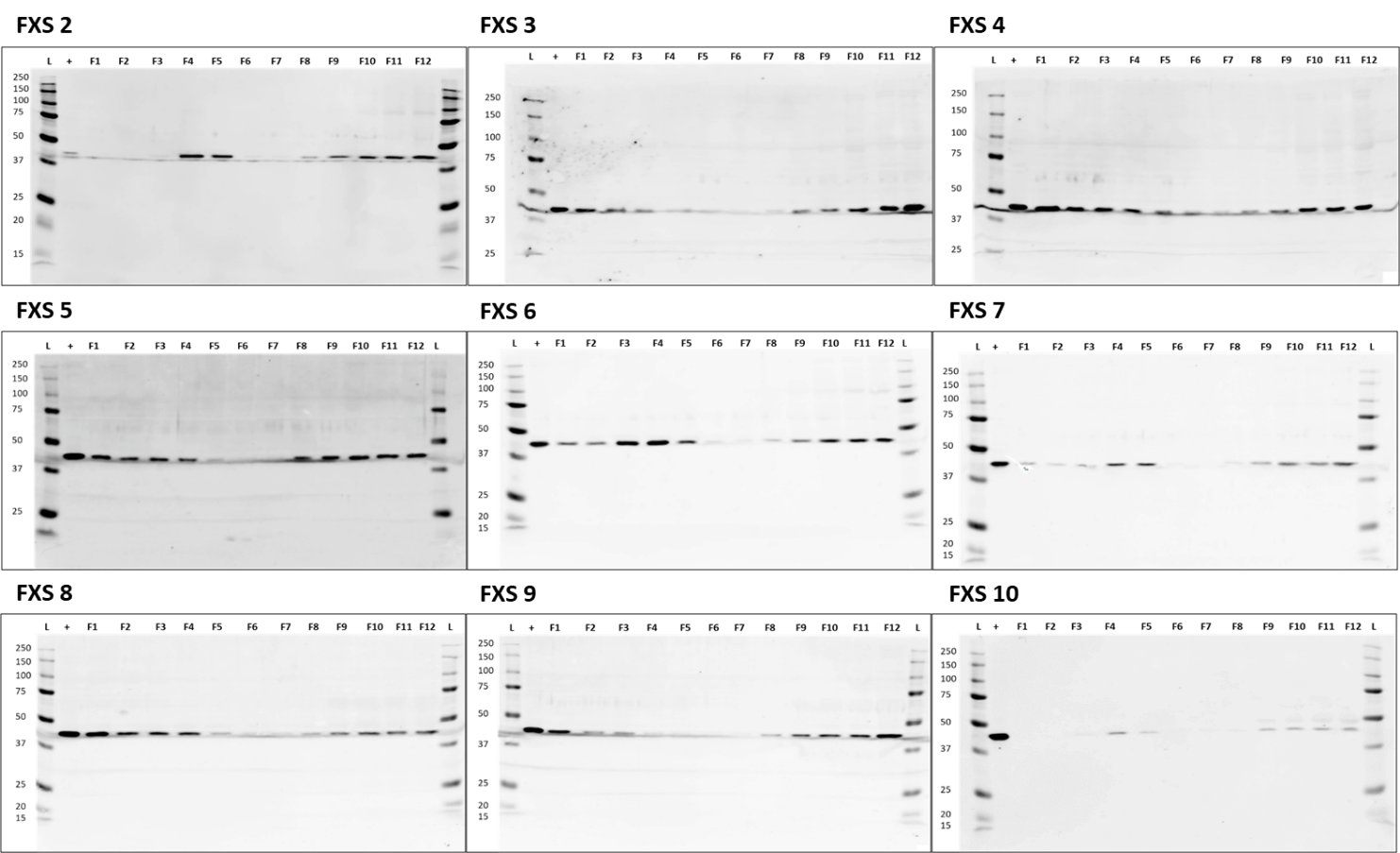


**
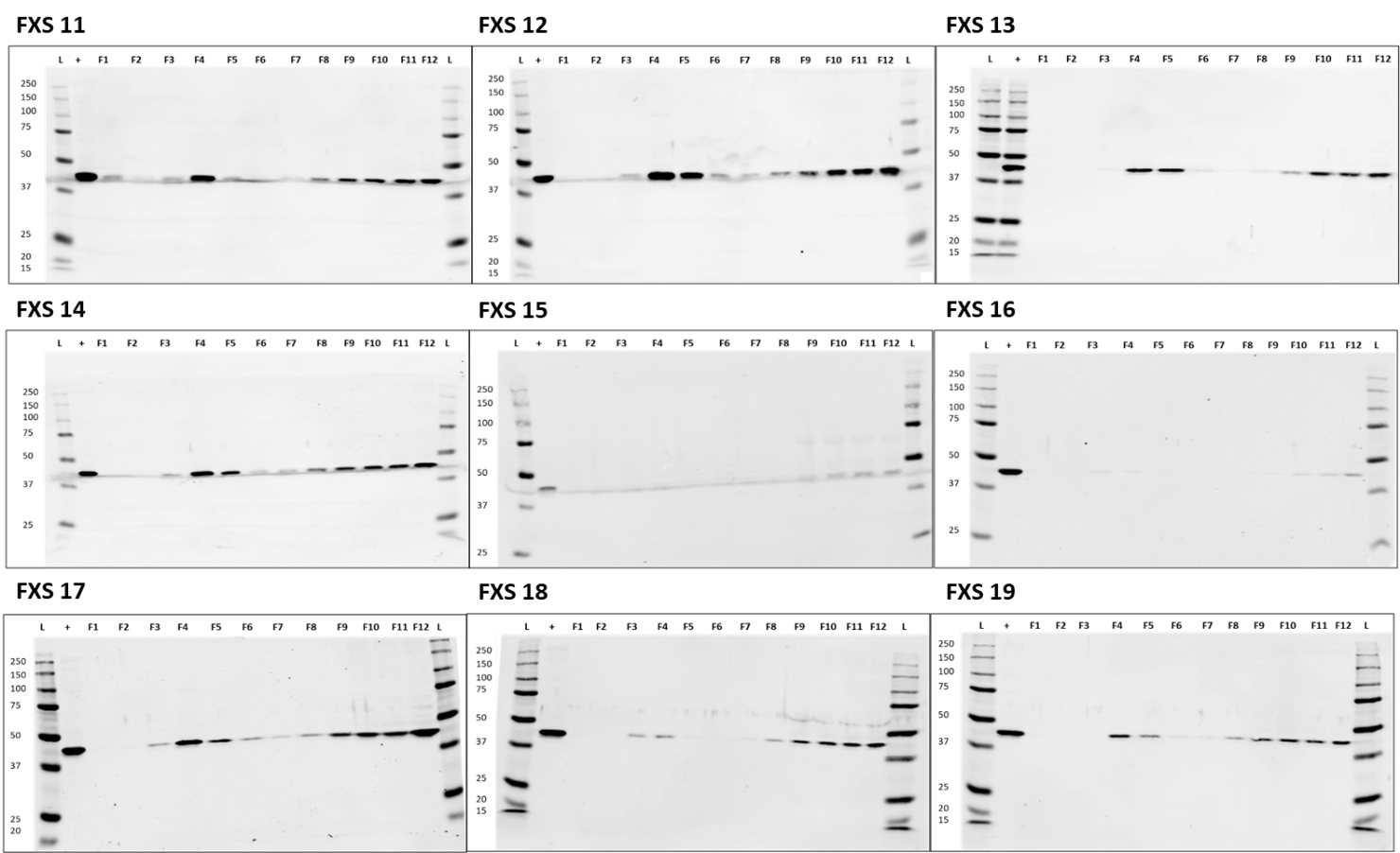
**

**
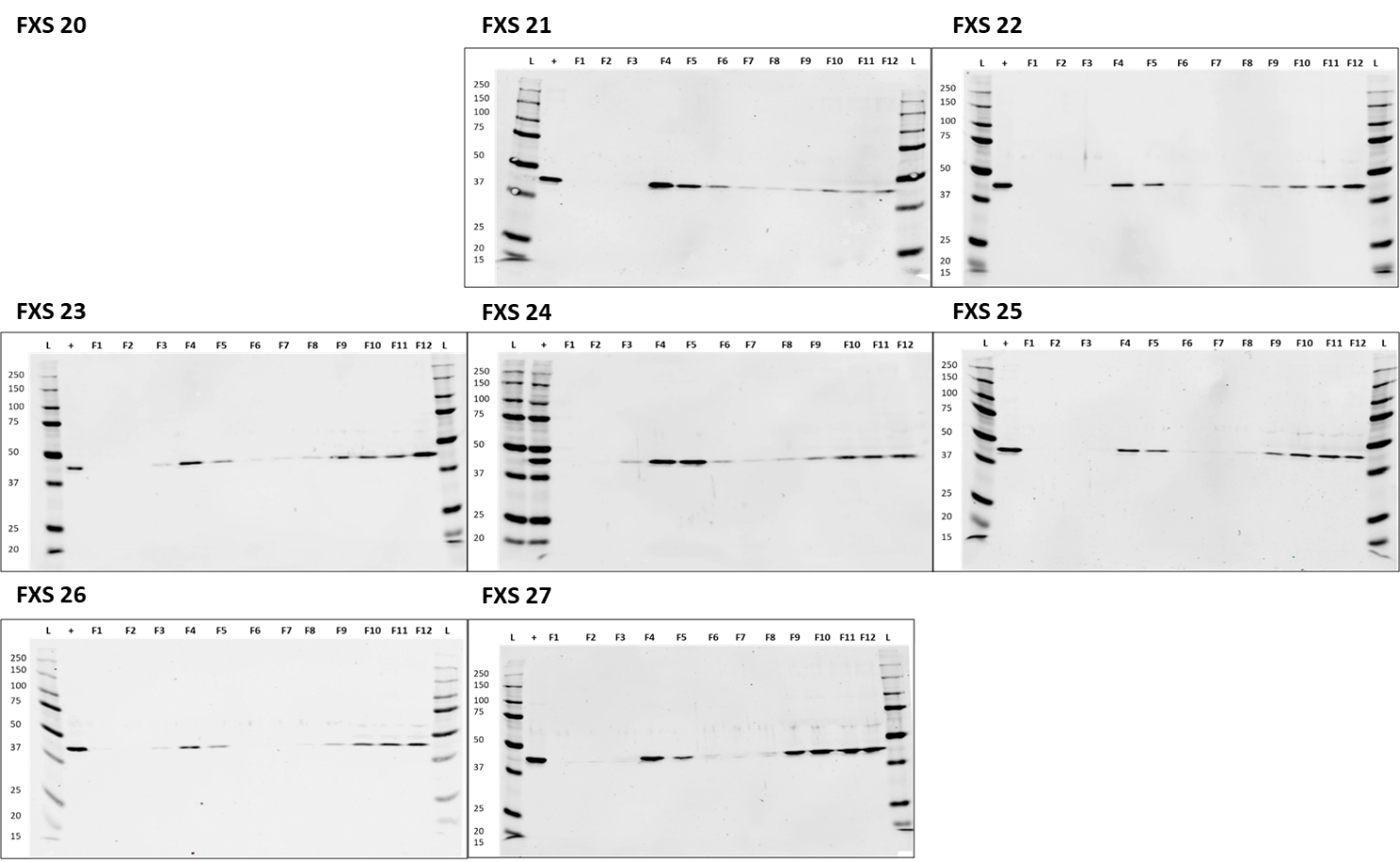
**

**Supplementary Figure 2.** Full length blots of Flotillin-1 for each participant controls and FXS.

L: ladder (kDa); +: loading control; F1-F12: fraction separated by sucrose gradient.

**
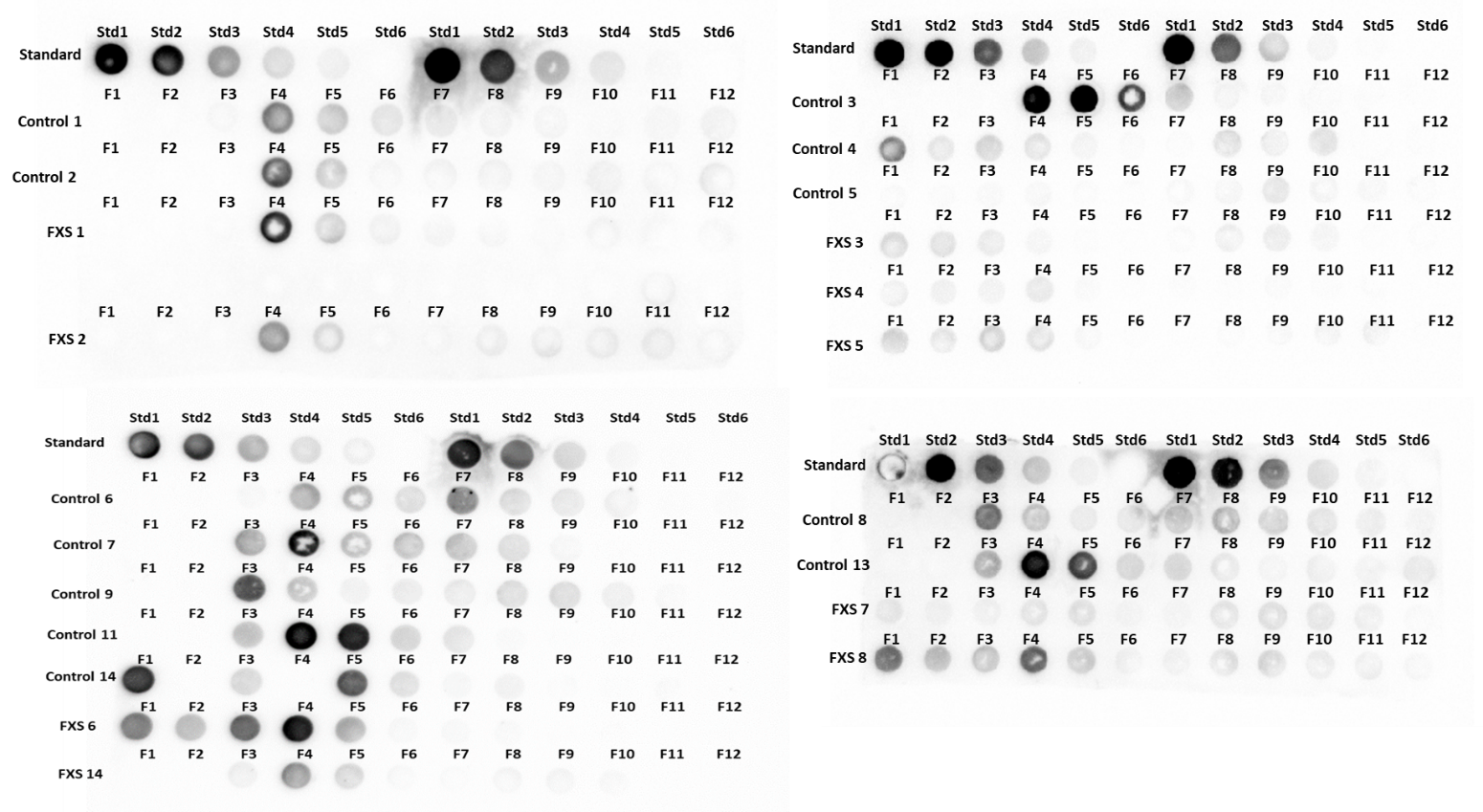
**

**
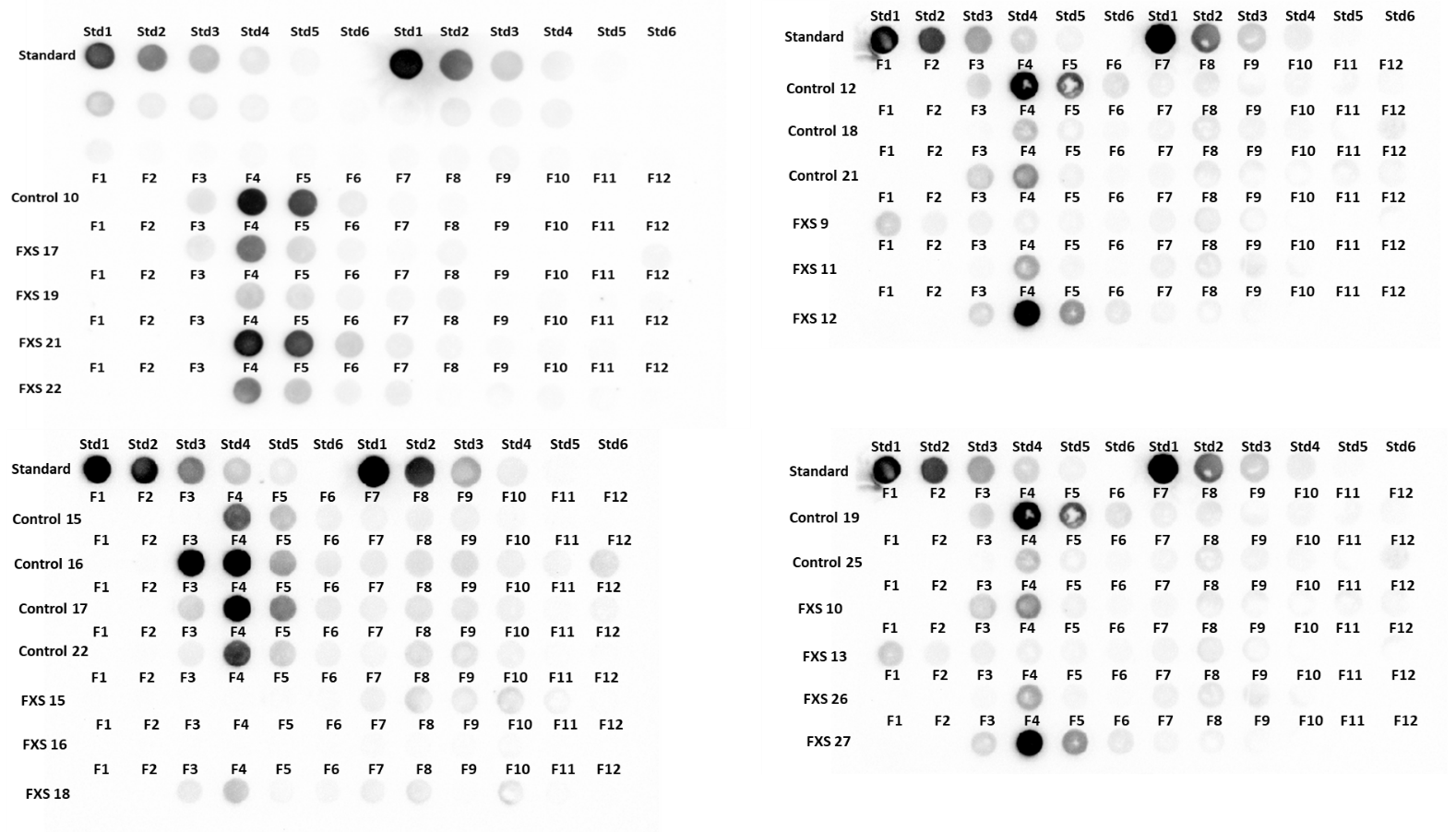
**

**
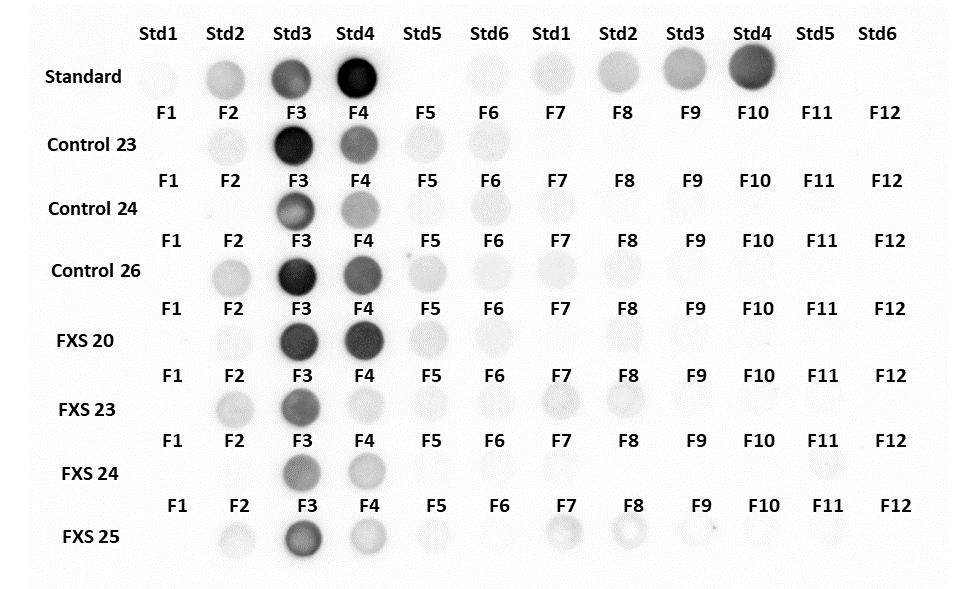
**

**Supplementary Figure 3.** Full length dot blots of ganglioside GM1 for each participant controls and FXS.

STD1-STD6: standard solution at 5.000; 2.500; 1.250; 0.625; 0.313 and 0.000 ng/mL

F1-F12: fraction separated by sucrose gradient

**Supplementary Table 2.** Coefficient of determination of simple and multiple linear regression models adjusted for either age, BMI or FMRP expression (absence or presence).

|  | **Model 1**  **(LR chol.)** | | **Model 2**  **(LR chol. + age)** | | **Model 3**  **(LR chol. + BMI)** | | **Model 4**  **(LR chol. + FMRP)** | |
| --- | --- | --- | --- | --- | --- | --- | --- | --- |
|  | **R^2^** | ***p*-value** | **R^2^_adj_** | ***p*-value** | **R^2^_adj_** | ***p*-value** | **R^2^_adj_** | ***p*-value** |
| **ABAS total** | 0.48 | < 0.001 | 0.48 | 0.001 | 0.44 | 0.002 | 0.51 | 0.014 |
| ABAS conceptual | 0.45 | < 0.001 | 0.42 | 0.001 | 0.40 | 0.002 | 0.54 | 0.015 |
| ABAS social | 0.44 | < 0.001 | 0.40 | 0.002 | 0.41 | 0.007 | 0.48 | 0.017 |
| ABAS practical | 0.39 | 0.002 | 0.41 | 0.005 | 0.33 | 0.007 | 0.37 | 0.025 |
| **SCQ** | 0.45 | < 0.001 | 0.42 | 0.002 | 0.40 | 0.005 | 0.62 | 0.020 |

R^2^_adj_: coefficient of determination adjusted for multiple predictors
